# Gender specific effect of LIPC C-514T polymorphism on obesity and relationship with plasma lipid levels in Chinese children

**DOI:** 10.1111/jcmm.12663

**Published:** 2015-08-18

**Authors:** Hao Wang, Dandan Zhang, Jie Ling, Wenhui Lu, Shuai Zhang, Yimin Zhu, Maode Lai

**Affiliations:** a Department of Pathology, Zhejiang University School of MedicineHangzhou, Zhejiang, China; b Key Laboratory of Disease Proteomics of Zhejiang ProvinceHangzhou, Zhejiang, China; c Department of Epidemiology & Biostatistics, Zhejiang University School of Public HealthHangzhou, Zhejiang, China

**Keywords:** polymorphism, obesity, lipids, children

## Abstract

Hepatic lipase (LIPC) is a key rate-limiting enzyme in lipoprotein catabolism pathways involved in the development of obesity. The C-514T polymorphism in the promoter region is associated with decreased LIPC activity. We performed a case-controlled study (850 obese children and 2119 controls) and evaluated the association between LIPC C-514T polymorphism, obesity and plasma lipid profile in Chinese children and adolescents. Additionally, we conducted a meta-analysis of all results from published studies as well as our own data. A significant association between the polymorphism and obesity is observed in boys (*P* = 0.042), but not in girls. And we observed a significant relationship of the polymorphism with total cholesterol (TC) and high density lipoprotein cholesterol (HDL-C) independent of obesity in boys. The T allele carriers have higher levels of low density lipoprotein cholesterol (LDL-C) in obese boys, and triglyceride (TG), TC and LDL-C in non-obese girls (all *P* < 0.05). In the meta-analysis, under dominant model the T allele increased body mass index (BMI) level in boys, while it decreased BMI in girls, and increased the levels of TC both in the overall and subgroups, TG and HDL-C in the overall and boys, and LDL-C in the overall (all *P* < 0.05). Our results suggest that the T allele might carry an increased risk of obesity in Chinese boys. The meta-analysis suggests that T allele acts as a risk allele for higher BMI levels in male childhood, while it is a protective allele in female childhood. And the polymorphism is associated with the levels of plasma lipids, which may be modulated by obesity and gender.

## Introduction

The hepatic lipase gene (LIPC) is a member of the lipase gene family, in which protein sequence homology with other lipases is 30–75% 1. LIPC has been cloned from two human liver cDNA libraries by cross-hybridization with the determined rat cDNA clone 2. It is a secretory glycoprotein enzyme synthesized predominantly by the liver, and distributed on the surfaces of hepatocytes and sinusoidal endothelium 3.

Since it plays an important role in lipoprotein catabolism pathways 3, LIPC has been implicated in the risk of coronary artery disease, where its effect is dependent on the underlying lipoprotein phenotype or disorder 4. Hepatic lipase also plays a role in energy homeostasis. LIPC was identified as an obesity candidate gene in a mouse model, LIPC deficient mouse can be protected against diet-induced obesity 5. And associations were observed between the LIPC polymorphisms and body mass index (BMI) in humans 6–8. As reported, human hepatic lipase activity increased with increasing visceral adiposity, whereas loss of intra-abdominal fat was associated with reduced hepatic lipase activity 5. And the hepatic lipase activity is influenced by genetic variation, gender, intracellular cholesterol, lipid-lowering therapy, BMI and intra-abdominal fat deposition 8,9. According to the previous studies, genetic variation accounted for 30–45% of the variation in hepatic lipase activity 10. LIPC C-514T polymorphism in the promoter region has attracted considerable attention, in which 514T allele confers a decreased synthesis and activity in hepatic lipase 10,11.

The role of LIPC C-514T polymorphism in high density lipoprotein cholesterol (HDL-C) production has been well-established 12,13, but the association of the polymorphism with obesity is less certain. What’s more, numerous studies focusing on the relationship between the polymorphism and various disorders have been carried out in adults, but less attention has been placed on children and adolescents. So, in the present study, we have reevaluated the relationship between the C-514T polymorphism and obesity and the potential interactions of C-514T polymorphism with gender and obesity on plasma lipid profile in Chinese children and adolescents.

## Materials and methods

### Study subjects

The present study included 2969 unrelated subjects (850 cases, 2119 controls), aged 7–17 years old. The subjects were recruited from a cross-sectional study on metabolic syndrome of children and adolescents conducted in six regions in China (Beijing, Shanghai, Hangzhou, Tianjin, Chongqing and Nanning) in 2010. The BMI classification reference recommended by the Working Group on Obesity in China in 2004 was applied to serve as the criteria for obesity 14. All subjects were free of cardiac, pulmonary, hepatic and renal disorders, or other serious diseases. The study was approved by the Research Ethics Committees of the School of Public Health and the Medical Ethics Committees of the Children’s Hospital of the College of Medicine, Zhejiang University.

### Clinical characteristics

Anthropometric measurements including weight, and height were examined by the trained investigators and the children were asked to be lightly dressed and barefoot. Body mass index was calculated as weight divided by the square of height (kg/m^2^). Clinical parameters included measurement of the concentrations of triglyceride (TG), total cholesterol (TC), low density lipoprotein cholesterol (LDL-C) and HDL-C in the plasma.

### SNP genotyping

Human genomic DNA was extracted from the peripheral blood leukocytes using the TOYOBO MagExtractor Genomic DNA Purification Kit (Toyobo, Osaka, Japan). After extraction, the DNA samples were diluted to a final concentration of 20 ng/μl and the genotype of the C-514T polymorphism was performed using the Sequenom MassARRAY iPLEX platform 15.The amplification primers were 5′-ACGTTGGATGAGGGCATCTTTGCTTCTTCG-3′ and 5′-ACGTTGGATGAAGTGTGGTGCAGAAAACCC-3′, and the extension primer was 5′-AAAAACCCTTCACCCCC-3′. The experimental operations and MassARRAY data analysis were carried out by the Bio-X Institutes, Shanghai, China. Quality control procedures of genotyping consisted of a >95% successful call rate, duplicate calling of genotypes and internal positive control samples.

### Statistical analysis

The clinical characteristics of continuous values were presented as a mean with S.D., and the polymorphism genotype frequency was presented as a number with percentage according to case**–**control status. The Hardy**–**Weinberg equilibrium (HWE) was assessed using the chi-squared goodness-of-fit test. Independent *t*-tests and chi-squared tests were used for comparisons of means and proportions between cases and controls.

Logistic regression was applied to test the associations of LIPC C-514T polymorphism with obesity adjusted for age and stratified by gender. The trend tests were analyzed by chi-squared test. A multiple linear regression model was applied to estimate the associations of LIPC C-514T polymorphism with plasma TG, TC, LDL-C and HDL-C, and their interactions modified with age and stratified by obesity status and gender. The analysis was performed using a stepwise approach.

All the statistical analysis was performed using SPSS 20.0 for Windows (IBM Corp, Armonk, NY, USA). A two-sided *P* < 0.05 was considered statistically significant.

### Meta-analysis

A comprehensive literature search was conducted for relevant publications covering the period up to December 2014, from the sites PubMed, MEDLINE and ISI Web of Knowledge. Search strategies included keywords as ‘LIPC’, or ‘HL’, or ‘hepatic lipase’, and ‘polymorphism’, or ‘variant’, or ‘SNP’, and ‘children’ or ‘adolescents’. The publications were limited to English, and those that examined the relationship between the LIPC C-514T polymorphism and BMI, or plasma lipid levels according to gender in children and adolescents were included in the meta-analysis. The duplicated papers were excluded if the subjects were from the same population, only the research with a wealth of information was included. And the studies were excluded if the indices of outcome were not able to be extracted or estimated from the published paper or by contacting the authors. The following information was extracted from each study, including first author name, publication year, region/ethnicity composition of the population, age, method of genotyping, sample size, genotype frequencies and mean with S.D. or standard error of BMI and serum lipid parameters by genotypes.

The associations of the polymorphism with BMI and lipid parameters were estimated by weighted mean difference (WMD) and its 95% confidence interval (CI) stratified by gender under a dominant model (CT/TT *versus* CC). The heterogeneity among the studies was quantified by using an inconsistency index (*I*^2^), and confirmed significant with the *P*-value less than 0.1. A fixed-effect model was applied with no heterogeneity existing, otherwise, the random-effect model was adopted. The publication bias was assessed by Begg’s funnel plot and the Eegg’s linear regression. The *P*-value for the HWE test was calculated by a goodness-of-fit chi-squared test in the participants for each study. The meta-analysis was conducted using STATA software (v.12.0 for windows; Stata Corporation, College Station, TX, USA). A two-sided *P* < 0.05 was considered statistically significant.

## Results

Table1 summarizes the anthropometric and biochemical characteristics of the participants according to case**–**control status. Comparisons between case and control group showed several variables have statistically significant differences, with the exceptions of gender distribution (*P* = 0.443). In contrast with the control group, the case group tended to be younger, and had higher levels of anthropometric indexes (height, weight), plasma lipid concentrations (TG, TC and LDL-C), while lower HDL-C levels (all *P* < 0.001; Table1).

**Table 1:** Anthropometric, biochemical and genetic characteristics of the subjects

Characteristics	Obesity (*N* = 850)	Normal weight (*N* = 2119)	*P*-value
Age (years)	11.2 ± 2.8	11.6 ± 2.5	<0.001*
Gender, *n* (%)			
Boys	565 (66.5)	1376 (64.9)	0.443†
Girls	285 (33.5)	743 (35.1)	
Height (cm)	150.5 ± 15.7	146.9 ± 16.2	<0.001*
Weight (kg)	62.4 ± 20.0	38.0 ± 11.9	<0.001*
TG (mmol/l)	1.23 ± 0.63	0.78 ± 0.36	<0.001*
TC (mmol/l)	4.23 ± 0.79	3.87 ± 0.72	<0.001*
LDL-C (mmol/l)	2.40 ± 0.66	1.96 ± 0.54	<0.001*
HDL-C (mmol/l)	1.33 ± 0.40	1.51 ± 0.38	<0.001*

* Independent *t*-test.

† Chi-squared test.

Continuous variables are presented as mean ± S.D.

TG: triglyceride; TC: total cholesterol; LDL-C: low density lipoprotein cholesterol-cholesterol; HDL-C: high density lipoprotein cholesterol.

As shown in Table2, the genotype frequencies for LIPC C-514T polymorphism were 36.1% for CC homozygote, 47.9% for CT heterozygote and 16.0% for TT homozygote in the case group; 39.2% for CC, 46.9% for CT and 13.9% for TT in the control group. Genotype distributions were consistent with the HWE among controls (*P* = 0.91). But the distribution of the genotype between the case and control group did not achieve statistical significance (*P* = 0.069). After the data were stratified by gender, the polymorphism was associated significantly with obesity in boys (CT *versus* CC: OR = 1.11, 95% CI = 0.93–1.32; TT *versus* CC: OR = 1.24, 95% CI = 0.97–1.58; *P* = 0.042), and the trend test showed a significant effect of the polymorphism on obesity (*P* = 0.038). However, no positive association was observed in girls. A trend was found in the whole subjects and the boy’s subgroup in that the BMI level increased with the number of T allele, however, only marginal significant results were found under the additive model. And neither trend nor statistical significance was observed in the girl’s subgroup (Table S1).

**Table 2:** Association between LIPC C-514T genotype and obesity

Genotypes	Obesity, *n* (%)	Normal weight, *n* (%)	OR (95%CI)	*P* *
All				
CC	307 (36.1)	831 (39.2)	1	0.069
CT	407 (47.9)	994 (46.9)	1.11 (0.93–1.32)	
TT	136 (16.0)	294 (13.9)	1.24 (0.97–1.58)	
*P* for trend†	0.061			
Boys				
CC	200 (35.4)	549 (39.9)	1	0.042
CT	272 (48.1)	637 (46.3)	1.17 (0.94–1.45)	
TT	93 (16.5)	190 (13.8)	1.34 (0.99–1.80)	
*P* for trend†	0.038			
Girls				
CC	107 (37.5)	282 (38.0)	1	0.784
CT	135 (47.4)	357 (48.0)	1.01 (0.75–1.36)	
TT	43 (15.1)	104 (14.0)	1.07 (0.70–1.63)	
*P* for trend†	0.752			

* *P*-value was calculated by logistic regression adjust for age.

† *P*-value for trend was calculated by chi-squared test.

In order to further explore the effect of C-514T polymorphism on plasma lipid levels, the whole population was stratified by obesity status and gender under the dominant model. The T allele carriers in obese boys had significantly higher levels of TC, LDL-C and HDL-C, while similar relationships were observed in non-obese boys with TC and HDL-C (all *P* < 0.05). Significant associations of the T allele with higher levels of TG, TC and LDL-C were only found in normal weight girls compared with CC homozygote (all *P* < 0.05). And significant interaction between the genotype and gender for the TG level was observed in the normal weight individuals, the girls with the T allele had the highest level of TG compared with the other groups (*P* = 0.048; Table3).

**Table 3:** Stratified analysis of LIPC C-514T genotype and gender in association with plasma lipid concentrations by obesity

Variables	Normal weight		*P*	*P* for interaction	Obesity		*P*	*P* for interaction
	CC	CT/TT			CC	CT/TT		
TG (mmol/l)								
Boys	0.74 ± 0.38	0.75 ± 0.32	0.804	0.048	1.23 ± 0.64	1.24 ± 0.68	0.821	0.247
Girls	0.81 ± 0.32	0.88 ± 0.39	0.012		1.18 ± 0.49	1.28 ± 0.63	0.111	
TC (mmol/l)								
Boys	3.74 ± 0.64	3.83 ± 0.68	0.010	0.540	4.12 ± 0.77	4.29 ± 0.74	0.015	0.894
Girls	3.92 ± 0.72	4.06 ± 0.81	0.026		4.11 ± 0.84	4.29 ± 0.88	0.097	
LDL-C (mmol/l)								
Boys	1.86 ± 0.48	1.90 ± 0.49	0.171	0.162	2.32 ± 0.57	2.44 ± 0.62	0.028	0.640
Girls	2.04 ± 0.53	2.14 ± 0.64	0.022		2.30 ± 0.71	2.46 ± 0.79	0.073	
HDL-C (mmol/l)								
Boys	1.47 ± 0.35	1.53 ± 0.38	0.001	0.153	1.26 ± 0.34	1.34 ± 0.39	0.011	0.260
Girls	1.52 ± 0.37	1.53 ± 0.38	0.647		1.34 ± 0.48	1.38 ± 0.44	0.758	

Continuous variables are presented as mean ± S.D.

*P*-value was calculated in the linear regression model adjusted for age.

TG: triglyceride; TC: total cholesterol; LDL-C: low density lipoprotein cholesterol-cholesterol; HDL-C: high density lipoprotein cholesterol.

Table4 summarizes the characteristics of studies included in the meta-analysis. Because of its significantly differed genotype and allele frequencies between blacks and whites, the study conducted by Chen *et al*. in 2003 was divided into two studies as American whites and blacks. No studies deviating from HWE with an exception of the boy group from the study of Riestra *et al*. in 2009. Together with our study, four studies with 8069 subjects (4109 boys and 3960 girls) were selected into the meta-analysis for the association of C-514T polymorphism with BMI. Three studies with 4555 subjects (2723 boys and 1832 girls) evaluated the polymorphism about plasma levels of TG and TC, and two studies with 3295 subjects (2089 boys and 1206 girls) evaluated the polymorphism about LDL-C concentration, and five studies with 8034 subjects (4086 boys and 3948 girls) evaluated the polymorphism about HDL-C concentration.

**Table 4:** Characteristics of the included studies in meta-analysis

Author	Year	Region/ethnicity	Age	Genotyping assay	Sample size	Genotype frequencies (%)			HWE *P*-value
					Boys/girls	CC	CT	TT	
Chen *et al*.	2003	American whites	4–17 years	TaqMan assay	969/1450	1481 (61.22)	817 (33.78)	121 (5.00)	0.54
Chen *et al*.	2003	American blacks	4–17 years	TaqMan assay	395/666	223 (21.02)	530 (49.95)	308 (29.03)	0.86
Riestra *et al*.	2009	Spain	6–8 years	Restriction fragment length polymorphism (RFLP)	634/626	762 (60.48)	456 (36.19)	42 (3.33)	0.01*
Agirbasli *et al*.	2013	Turkish	6–17 years	Restriction fragment length polymorphism (RFLP)	170/190	244 (67.78)	105 (29.17)	11 (3.05)	0.94
Present study	2010	Chinese	6–17 years	Sequenom MassARRAY iPLEX	1941/1028	1138 (38.33)	1401 (47.19)	430 (14.48)	0.97

* HWE *P*-value is 0.001 in boys and 0.55 in girls.

HWE: Hardy**–**Weinberg equilibrium.

**Figure 1:**
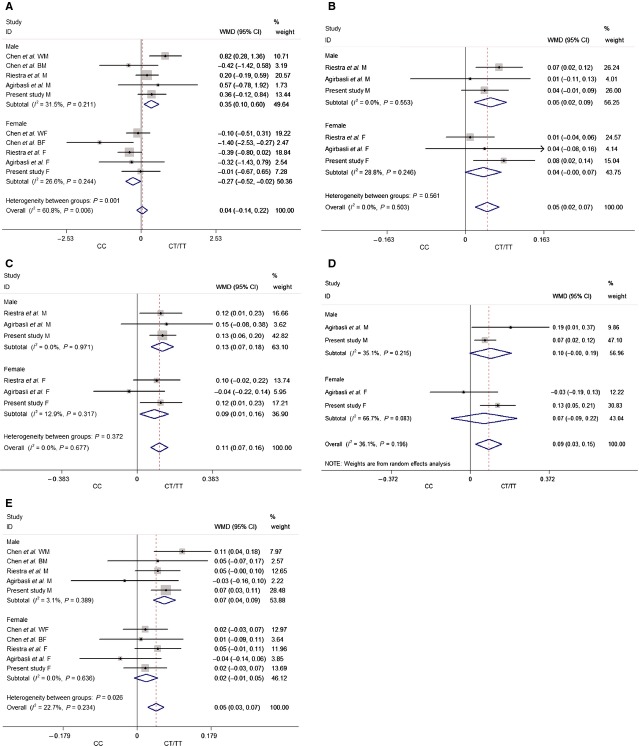
Forest plots of the association of LIPC C-514T polymorphism with BMI and plasma lipids. (A) BMI; (B) TG; (C) TC; (D) LDL-C; (E) HDL-C. BMI: body mass index; TG: triglyceride; TC: total cholesterol; LDL-C: low density lipoprotein cholesterol; HDL-C: high density lipoprotein cholesterol.

Under the fixed-effect model, the T allele carriers had an increased BMI level compared with the CC homozygote in the boy’s subgroup (WMD = 0.35, 95% CI = 0.10–0.60, *P* = 0.007; *I*^2^ = 31.5%, *P* = 0.211). However, the results in the girl’s subgroup showed an opposite effect trend since the T allele reduced the BMI level (WMD = −0.27, 95% CI = −0.52 to −0.02, *P* = 0.034; *I*^2^ = 26.6%, *P* = 0.244). The overall effect was weakened to non-significance due to the opposite direction effect on the subgroups (Fig.1A).

Under the fixed-effect model, the T allele carriers showed an increased TG concentration compared with the CC homozygote in the boy’s subgroup (WMD = 0.05, 95% CI = 0.02–0.09, *P* = 0.002; *I*^2^ = 0.0%, *P* = 0.553). The results in the girl’s subgroup showed a consistent trend while the differences did not reach a statistically significant level (WMD = 0.04, 95% CI = −0.00 to 0.07, *P* = 0.056; *I*^2^ = 28.8%, *P* = 0.246). And a significant increase in effect was also observed in the overall (WMD = 0.05, 95% CI = 0.02–0.07, *P* < 0.001; *I*^2^ = 0.0%, *P* = 0.503) (Fig.1B).

In the analysis with TC, similar results were found in the boy’s and girl’s subgroups and the overall, the T allele carriers showed an increased TC concentration compared with the CC homozygote under the fixed-effect model [WMD = 0.13, 95% CI = 0.07–0.18, *P* < 0.001 (*I*^2^ = 0.0%, *P* = 0.971); WMD = 0.09, 95% CI = 0.01–0.16, *P* = 0.002 (*I*^2^ = 12.9%, *P* = 0.317); WMD = 0.11, 95% CI = 0.07–0.16, *P* < 0.001 (*I*^2^ = 0.0%, *P* = 0.677), respectively] (Fig.1C).

Since significant heterogeneity was observed in the boy’s subgroup (*I*^2^ = 66.7%, *P* = 0.083), the analysis with LDL-C was conducted using the random-effect model. The T allele significantly increased the LDL-C concentration compared with the CC homozygote in the overall without heterogeneity (WMD = 0.09, 95% CI = 0.03–0.15, *P* = 0.005; *I*^2^ = 36.1%, *P* = 0.196). Although similar trend effects were also observed in the boy’s and girl’s subgroups, the tendency failed to achieve statistical significance [WMD = 0.10, 95% CI = −0.00 to 0.19, *P* = 0.053 (*I*^2^ = 35.1%, *P* = 0.215); WMD = 0.07, 95% CI = −0.09 to 0.22, *P* = 0.405, respectively] (Fig.1D).

Under the fixed-effect model, the T allele carriers showed an increased HDL-C concentration compared with the CC homozygote in the boy’s subgroup (WMD = 0.07, 95% CI = 0.04–0.09, *P* < 0.001; *I*^2^ = 3.1%, *P* = 0.389). And a consistent trend was also observed in the girl’s subgroup while no significance was detected (WMD = 0.02, 95% CI = −0.01 to 0.05, *P* = 0.131; *I*^2^ = 0.0%, *P* = 0.636). The result of the overall was similar with that in the boy’s subgroup (WMD = 0.05, 95% CI = 0.03–0.07, *P* < 0.001; *I*^2^ = 22.7%, *P* = 0.234; Fig.1E).

**Figure 2:**
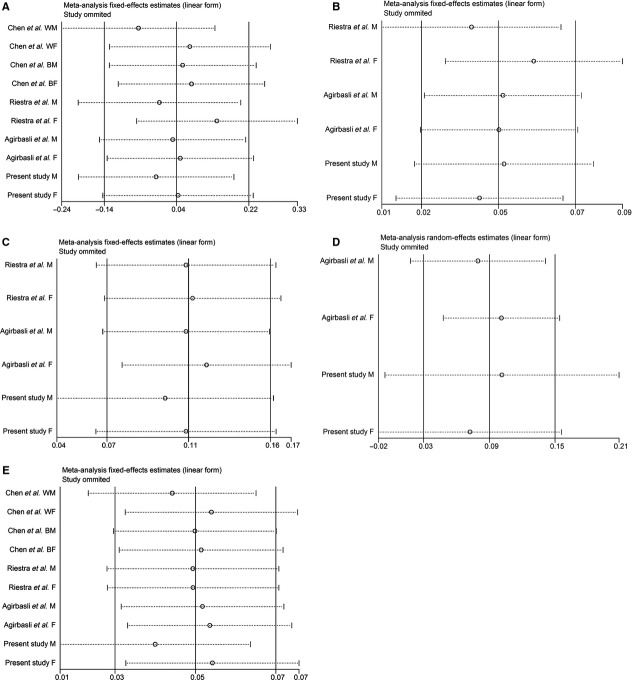
Sensitivity analysis for the association of LIPC C-514T polymorphism with BMI and plasma lipids. (A) BMI; (B) TG; (C) TC; (D) LDL-C; (E) HDL-C. See Figure1 for abbreviations.

A sensitivity analysis was conducted by omitting one study at a time and recalculating the pooled WMD for the remaining studies. The results suggest that no individual studies substantially influenced the pooled point estimate (Fig.2).

**Figure 3:**
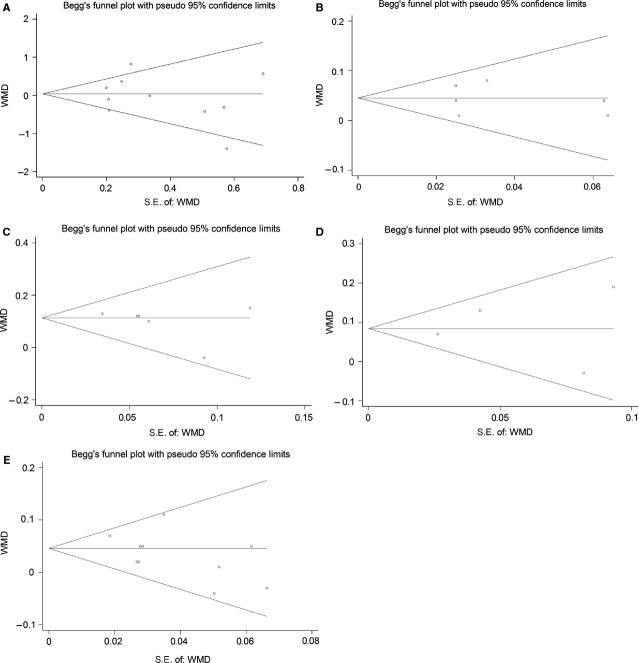
Begg’s funnel plots of the association of LIPC C-514T polymorphism with BMI and plasma lipids. (A) BMI; (B) TG; (C) TC; (D) LDL-C; (E) HDL-C. See Figure1 for abbreviations.

Begg’s funnel plot and Eegg’s linear regression test were performed to estimate the potential publication bias of the included studies. As shown in Figure3, no obvious asymmetry was found in the funnel plots, which was supported by the Egger’s test (all *P* > 0.05; Fig.3).

## Discussion

In the present study, we explored the associations between the LIPC C-514T polymorphism, obesity and plasma lipid profiling in a representative sample of Chinese children and adolescents. We found a significant relationship between the LIPC C-514T polymorphism and obesity in boys. The T allele increased the risk of obese boys. No significant association was observed in girls. The meta-analysis yielded similar results in boys in that the T allele increased the risk of obesity. While a significant opposite effect was revealed by the meta-analysis in girls. Several studies have explored the relationship of LIPC C-514T polymorphism and obesity. Most of them failed to detect a positive association 6,16–21, and few confirm the association 7,8,22. Tai *et al*. 22 reported an additive effect of the polymorphism on BMI in a mixed gender population of Malays, and the TT homozygotes had a higher level of BMI than the CC and CT individuals. In a French-Canadian men’s population 8, the TT significantly increased the BMI level compared with the C allele carriers which was consistent with our results for boys. The Bogalusa Heart Study 7 found a significant association only in black females for both adulthood and childhood who had higher frequencies of T allele, yet the T allele conferred the reduced level of BMI which supported the results of meta-analysis in the girl’s subgroup. These results may suggest that gender, age and ethnic variation may be involved in the association between the C-514T polymorphism and obesity.

LIPC has been reported as a factor involved in the control of energy balance and body fat accumulation, consequently producing an effect on obesity in mice; elevated hepatic lipase activity favours obesity, while its deficiency might protect against obesity 5,23. In humans, increased activity of hepatic lipase was also found related to a higher central body fat both in men and women 9,24. Paradoxically, the T allele is associated with reduced hepatic lipase activity 10,25, whereas in our meta-analysis, the T allele was positively related to obesity in the boy’s subgroup. While the results in the girl’s subgroup, in the meta-analysis, was in line with the T allele protective effect against obesity. The mechanism underlying the discrepancy may be due to the differentially influence of the sex steroid hormones. The hepatic lipase activity increases with the degree of obesity until it comes to the apparent maximum level in both genders 26. Once reaching the maximum level, LIPC activity is 33% higher in males than in females with the same genotype 27. Estrogenic steroids suppress LIPC activity as well as reduce its expression by the effect on apo A-1 production 28,29. As a key activity determinant, the C-514T genotypic effect was reported to modulate the influence of obesity on LIPC activity 25. So taken together, C-514T polymorphism interacts with gender to influence the activity of LIPC which regulates the BMI level.

We also compared the plasma lipid levels among LIPC C-514T genotypes stratified by gender and obesity status. In the normal weight group, the T allele conferred increased levels of TC and HDL-C in boys and TG, TC and LDL-C in girls. In the obesity group, the T allele associated with increased TC, LDL-C and HDL-C was only found in boys. A great deal of previous studies has examined the association of LIPC C-514T polymorphism with plasma lipid profile, and most of them found significance in HDL-C 12,17,22,30 while some failed to confirm the association 8,31. As to TG, TC, LDL-C, more studies present non-significant results 16,32–34, and little significance for TG 21,35, TC 21,35,36 and LDL-C 36,37. And these results varied according to gender and ethnicity. What’s more, less studies have been performed regarding the relationship between the C-514T polymorphism and plasma lipids in children and adolescents, and the results did not reach a consensus 7,35,38. So we performed a meta-analysis to evaluate the genetic effect of the polymorphism on plasma lipids levels. Under the dominant model, we found significant results in the association with HDL-C in the overall and boy’s subgroup, with TC both in the overall and subgroups, and TG and HDL-C in the overall and boys and LDL-C in the overall subjects. The association with HDL-C has been well-documented, Ko *et al*. 39 proved that the polymorphism was related to the HDL-C level only in obese men, and some studies proved the relationship in lean men 8,40. And there are other researches in which significant associations were also found in females 7,17,21,32. Chen *et al*. 7 reported significant results in the participants, except in the white female childhood. And Ji *et al*. 36 reported the association only in CHD patients rather than the healthy controls. And T allele was believed to increase the HDL-C level of children participants in upper percentiles of skinfold thickness in another study 38. As to TG, significant associations were observed by Fan *et al*. 21 in female adults, and by Riestra *et al*. 35 in male children. With TC, the significant associations were reported by Fan *et al*. 21 in female adults, by Ji *et al*. 36 in healthy control males and by Riestra *et al*. 35 in male children. With LDL-C, the significant associations were reported by Ji *et al*. 36 in healthy control males and by Agirbasli *et al*. 37 in female children. The inconsistency of these results may be due to the widely differed allele frequency of the polymorphism among ethnic groups, since the T allele confers the decreased LIPC expression and activity, which is related to dyslipidaemia 10,11,34. And the sex steroid hormones differentially influence the metabolism of plasma lipids may contribute to this discrepancy. Additionally, the bias from the varied schemes of sampling strategies for research purposes cannot be excluded.

The interaction between the genotypes and gender was statistically significant for TG only in normal weight subjects. The results indicated that the genotypic effect on the TG level was gender and obesity status dependent which was higher in girls compared with boys, and the presence of obesity abolished the effect on TG for girls. The interaction was not observed in other studies.

The study was performed in a children and adolescents population, which made the study avoid the potential confounders that may influence the genotypic effect on plasma lipids with adults, for instance, severe obesity, smoking and alcohol. A meta-analysis was conducted by Isaacs *et al*. 30 in 2004, but only hepatic lipase activity and HDL-C levels were included. In our meta-analysis, we did not exclude the boy groups deviating from HWE 35 according to the generally agreed theory, because it may not benefit from excluding the studies with a departure from HWE by its limited impact 41. And the results of the meta-analysis did not obviously change after removing the boy group.

Several limitations of the present study should be taken into consideration. First, after excluding the study of Talmud *et al*. 38 without detailed information, only two proper studies were available enrolled in the meta-analysis with LDL-C levels, including our study, and a significant heterogeneity was observed in the girl’s subgroup which may bias the results. Second, we did not investigate the effect of physical activity and dietary intake which were reported in previous studies 22,27,40, however, the impact of these lifestyle factors have not reached a consensus 33,40, so our results may not be prominently influenced by these types of confounders. Still, we cannot rule out the impact of potential undetected confounders. Additionally, we only explored a single polymorphism in the LIPC gene and did not include other essential polymorphisms of plasma lipid profile related genes, which may be contributive to the modification of plasma lipids.

In conclusion, the genotype distributions of the LIPC C-514T polymorphism were related to obesity status in a gender-specified fashion, male childhood with T allele might have a higher trend to be obese, while the female childhood with the T allele might tend to be defended against obesity. And the T allele confers elevated concentrations of TG, TC, LDL-C and HDL-C which might be modulated by gender and obesity.

## Supporting Information

**Table S1** Association between LIPC C-514T genotype and BMI under three genetic models.
