# Supplementary material for: Gender specific effect of LIPC C-514T polymorphism on obesity and relationship with plasma lipid levels in Chinese children
Source: J Cell Mol Med. 2015 Aug 18;19(9):2296–306. doi: 10.1111/jcmm.12663 (PMC4568933; doi:10.1111/jcmm.12663)
Supplement: Supplementary file 1 [file jcmm0019-2296-sd1.doc]

Association between LIPC C-514T genotype and BMI under 3 genetic models

|  | BMI (mean±SD） | | |  |  |  |
| --- | --- | --- | --- | --- | --- | --- |
|  | CC | CT | TT | *P*_additive_ | *P*_dominant_ | *P*_recessive_ |
| All | 19.77±5.27 | 19.94±5.20 | 20.24±5.56 | 0.177^a^ | 0.168^a^ | 0.103^a^ |
| Boys | 19.83±5.22 | 20.12±5.41 | 20.41±5.61 | 0.082^b^ | 0.103^b^ | 0.173^b^ |
| Girls | 19.65±5.37 | 19.62±4.77 | 19.90±5.47 | 0.640^b^ | 0.968^b^ | 0.364^b^ |

^a^Comparisons among genotypes by ANOVA adjusted for age and gender.

^b^Comparisons among genotypes by ANOVA adjusted for age.
